# Nutrients and Functional Components of Medicine and Food Homology Substances on Antidepressant Effects: A Mechanism-Oriented Review

**DOI:** 10.3390/molecules31101727

**Published:** 2026-05-19

**Authors:** Yamin Zhang, Lei Wang, Chenxi Liu, Jingzhang Geng

**Affiliations:** Collaborative Innovation Center for Comprehensive Development of Biological Resources in Qinba Mountain Area of Southern Shaanxi, Qinba Provincial Key Laboratory of Biological Resources and Ecological Environment, Shaanxi Province Key Laboratory of Bio Resources, College of Biological Science and Engineering, Shaanxi University of Technology, Hanzhong 723000, China; 16693228653@163.com (Y.Z.); 18392121535@163.com (L.W.); 18829592624@163.com (C.L.)

**Keywords:** medicine and food homology, depression, pathogenesis, nutrients, active ingredients, modern technology

## Abstract

Depression is one of the most common mental disorders in modern society, and it has become a serious threat to human health. The limitations of existing antidepressant drugs have prompted people to turn to the multi-target, low-toxic side effects of natural products. This article reviews the conventional nutrients (omega-3 fatty acids, folic acid, and mineral elements) and functional active ingredients (flavonoids, polysaccharides, saponins, and terpenoids) in medicinal and food homologous substances (MFHs). They show antidepressant potential by regulating neurotransmitters, improving hypothalamic–pituitary–adrenal (HPA) axis function, promoting neuroplasticity, inhibiting neuroinflammation, regulating ferroptosis, and interfering with the gut–brain axis. In addition, this paper discusses the application prospects of modern technologies such as microbial fermentation and nano-delivery in improving the bioavailability of MFHs and product development. In summary, MFHs have potential application value in dietary intervention and adjuvant therapies for depression; in the future, randomized controlled clinical trials should be strengthened, and multi-omics technology should be combined to promote the development of precision products so as to provide a new perspective for the development of new antidepressant drugs.

## 1. Introduction

Depression is a common and high-risk mental disorder characterized by persistent emotional depression, decreased interest, slow thinking, and sleep disorders. It seriously affects the quality of life of patients and increases the risk of suicide, bringing a heavy burden to families and society. With the influence of multiple pressures in modern society, depression is becoming more and more common in people of all ages, and the incidence rate is increasing year by year. According to the World Health Organization (WHO), more than 350 million people worldwide suffer from depression, and it is predicted to rise to the top of the global burden of disease by 2030 [1]. In addition, the occurrence of depression also significantly increases the risk of other diseases, such as Alzheimer’s disease, Parkinson’s disease, and cardiovascular disease [2]. At present, commonly used antidepressant drugs such as 5-hydroxytryptamine reuptake inhibitors mainly play a role by regulating the levels of neurotransmitters, but they generally have problems such as slow onset and significant side effects. Therefore, natural plants with neuroprotective effects have become potential sources of drugs for effective prevention or treatment of depression.

In recent years, the role of diet and dietary active ingredients in the prevention and treatment of depression and other emotional disorders has attracted much attention and is regarded as an important class of controllable risk factors [3]. In this context, medicinal and food homologous substances (MFHs), that is, natural products that not only meet the nutritional needs of daily consumption in traditional Chinese medicine but can also be used as medicinal materials to regulate the body and prevent diseases, have gradually attracted more and more of researchers’ attention. The ‘homology’ means that the medicine and food come from the same source, in terms of material source, and have the same taste characteristics and efficacy. These substances have overall conditioning, multi-target effects, and neuroprotective potential. The theory of traditional Chinese medicine believes that depression can be caused by “Qi” (vital energy), blood stasis, “heat” (inflammation), moisture, and phlegm, resulting in dysfunction of viscera [4]. The core principle of treatment is ‘soothing liver and relieving depression, regulating qi movement’, which provides a traditional theoretical basis for the compatibility and application of medicinal and edible homologous substances. As the earliest theoretical classic of traditional Chinese medicine, *Huangdi Neijing* records the discussion about ‘fasting food is food and patients eat medicine’ and expounds upon the idea of the homology of food and medicine. Modern studies have further shown that MFHs are rich in a variety of nutrients, flavonoids, saponins, and other active ingredients, and they have unique advantages for improving depressive symptoms, delaying disease progression, and improving the quality of life of patients [5,6,7]. This article focuses on the pathogenesis of depression and summarizes the conventional nutrients and functional components with antidepressant activity in MFHs and their mechanism of action. The application prospects of microbial fermentation, nano-delivery, and multi-omics technology in the development of functional foods were discussed in order to provide research ideas and references for dietary intervention and product development.

## 2. Main Pathogenesis of Depression

The pathogenesis of depression is not completely clear. Genetic factors and childhood experiences are closely related to the risk of depression. Environmental factors such as trauma and stress in life may also induce depression [8]. At present, the widely accepted mechanisms of depression include neurotransmitter disorders, changes in neural plasticity, hypothalamic–pituitary–adrenal (HPA) axis dysfunction, inflammatory response, ferroptosis, and gut–brain axis dysfunction [9].

### 2.1. Monoamine Neurotransmitter Disorders

**Figure 1:**
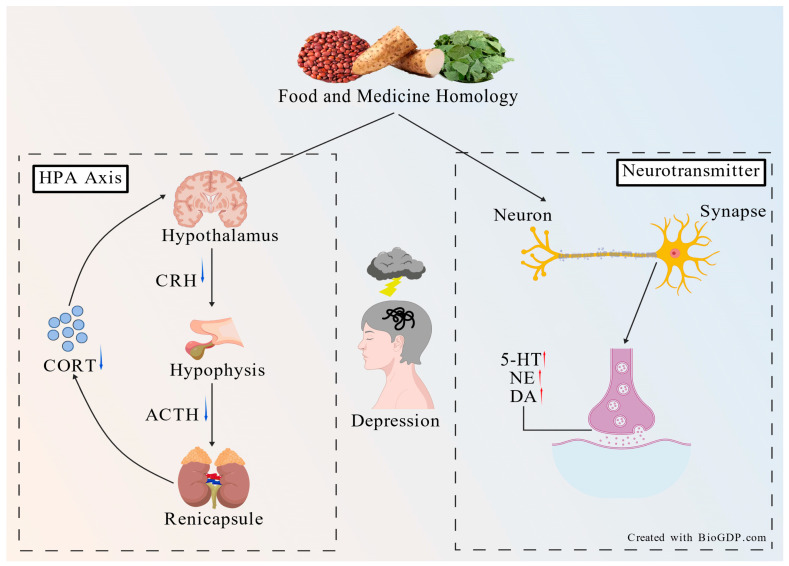
Antidepressant mechanism of MFHs targeting the HPA axis and neurotransmitters: (**left**) MFHs alleviate depression by reducing CRH, ACTH, and CORT levels; (**right**) MFHs alleviate depression by increasing levels of neurotransmitters such as 5-HT, NE, and DA.

The monoamine hypothesis is one of the traditional pathogeneses of depression. It is believed that the occurrence of depression is related to metabolic disorders of monoamine neurotransmitters, such as 5-HT, NE and DA, in the brain. It has a wide range of biological activities, involved in the regulation of emotion, sleep, cognition, and autonomic nervous function and other physiological processes [10]. Depression animal experiments and clinical studies have shown that the levels of 5-HT and NE and their metabolites and receptors in the body under stress are different from those in a normal state [11]. In addition, glutamate-induced activation of N-methyl-D-aspartate (NMDA) receptors can lead to calcium imbalance, resulting in impaired neuronal function, which is also one of the important pathological mechanisms of depression [12]. The mechanism of MFHs improving depression by interfering with neurotransmitters and their receptors is shown in Figure 1.

### 2.2. HPA Axis Disorder

The hypothalamic–pituitary–adrenal (HPA) axis is an important neuroendocrine system that regulates the body’s stress response. Studies have found that HPA axis function is overactivated in patients with depression, manifested by increased secretion of corticotropin-releasing hormone (CRH) from the hypothalamic paraventricular nucleus, which in turn stimulates adrenocorticotropic hormone (ACTH) release from the anterior pituitary and promotes glucocorticoid synthesis, leading to elevated serum corticosterone (CORT) levels [13]. Under physiological conditions, CORT inhibits CRH and ACTH release via negative feedback. Under stress, this negative feedback mechanism is weakened, resulting in HPA axis hyperfunction, which subsequently damages neuronal function, disrupts neurotransmitter metabolism, and reduces neurotrophic factor expression [14]. MFHs can improve depression by restoring the negative feedback balance of HPA axis, and its mechanism is shown in Figure 1.

### 2.3. Changes of Neural Plasticity

**Figure 2:**
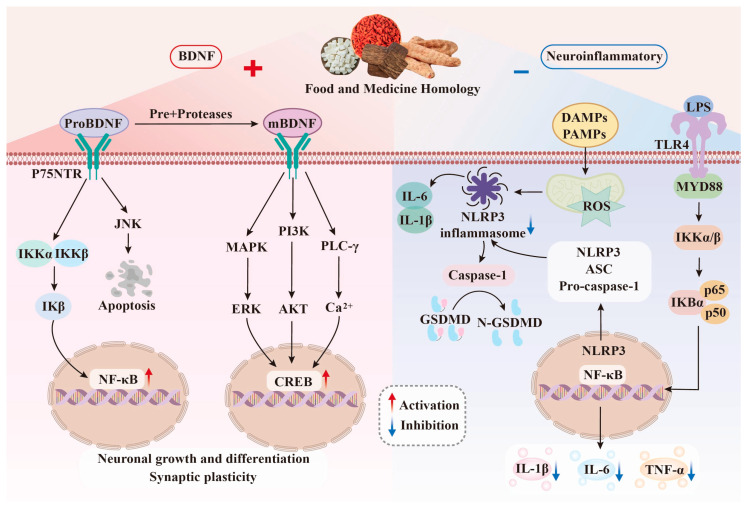
Antidepressant mechanism of MFHs targeted intervention on neurotrophic factors and inflammation: (**left**) pro-brain-derived neurotrophic factor (proBDNF) is partially cleaved into mature BDNF (mBDNF), which promotes neuronal growth, differentiation, and synaptic plasticity by activating MAPK/ERK, PI3K/AKT, and PLC-γ pathways. The combination of proBDNF and p75 neurotrophin receptor (p75NTR) induces nuclear factor-κB (NF-κB) activation and apoptosis; (**right**) pathogen-associated molecular patterns (PAMPs) and damage-associated molecular patterns (DAMPs) activate the TLR4/NF-κB pathway, induce NLRP3 inflammasome assembly, produce IL-1β, and trigger GSDMD-mediated pyroptosis, while upregulating TNF-α and IL-6. MFHs can enhance BDNF levels and reduce neuroinflammatory response.

Neuroplasticity refers to the ability of the nervous system to adapt to new situations by changing synaptic connections and neuronal reorganization due to injury or environmental stimulation. It is one of the important mechanisms for the brain to maintain cognitive and emotional functions. In depression, patients often show hippocampal atrophy, decreased dendritic spine density, and decreased neurogenesis [15]. Among them, brain-derived neurotrophic factor (BDNF) plays an important role. BDNF binds to the corresponding membrane receptors and activates downstream signaling pathways, such as phosphoinositide 3-kinase/protein kinase (PI3K/AKT), mitogen-activated protein kinase/extracellular regulated protein kinase (MAPK/ERK), and phospholipase C-γ (PLC-γ) to promote neuronal growth, differentiation and synaptic plasticity [16]. Clinical studies have shown that the level of BDNF in the brain of patients with depression is significantly lower than that of normal people [17]. In animal experiments, chronic stress and other pro-depression conditions can downregulate the expression of BDNF in the hippocampus, leading to increased neuronal apoptosis and weakened regeneration ability [18]. Therefore, regulating BDNF levels is expected to improve the pathological state of depression and promote the recovery of neuroplasticity. The mechanism of MFHs improving depression by regulating neurotrophic factors is shown in Figure 2.

### 2.4. Neuroinflammation

Neuroinflammation is a pathological process characterized by chronic inflammatory response in the central nervous system. As the main immune cells of the central nervous system, moderate activation of microglia can remove cell debris and damaged neurons, and promote cell survival, while excessive activation will secrete a large number of cytokines and chemokines, such as tumor necrosis factor-α (TNF-α), interleukin-1β (IL-1β), and interleukin-6 (IL-6) [19]. Studies have consistently found that patients with depression exhibit increased concentrations of inflammatory cytokines in the brain and peripheral blood, as well as microglial activation [20]. A number of meta-analyses have confirmed that IL-1β, IL-6, TNF-α, and C-reactive protein (CRP) in peripheral blood are inflammatory markers for patients with depression [21]. The expression of these inflammatory factors is regulated by Toll-like receptor 4 (TLR4) and its downstream nuclear factor-κB (NF-κB) and MAPK signaling pathways. Excessive production can cause neuronal damage, neurotransmitter imbalance, and HPA axis activation, thereby exacerbating depression [22]. In addition, NLRP3 inflammasome is another key regulator of neuroinflammation. Blocking NLRP3 inflammasome can improve the increase in IL-1β in peripheral blood and brain of mice induced by stress, and eliminate the depression-like behavior of mice [23]. MFHs can improve depression by inhibiting the TLR4/NF-κB/NLRP3 pathway and reducing pro-inflammatory factors, as shown in Figure 2.

### 2.5. Iron Death

**Figure 3:**
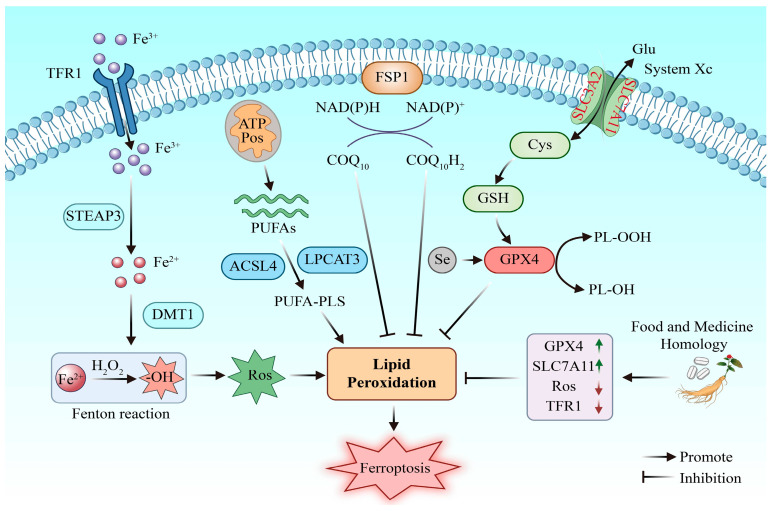
Antidepressant mechanism of MFHs targeted intervention in ferroptosis. After Fe^3+^ entered the cells through transferrin receptor 1 (TFR1)/six-transmembrane epithelial antigen of prostate 3 (STEAP3)/divalent metal transporter 1 (DMT1), reactive oxygen species (ROS) were produced by Fenton reaction. ACSL4 and LPCAT3 mediate the integration of polyunsaturated fatty acids (PUFAs) into membrane phospholipid PUFA-PLS to drive lipid peroxidation. GPX4 uses glutathione (GSH) to reduce toxic phospholipid peroxide (PL-OOH), while ferroptosis inhibitor protein 1 (FSP1) captures free radicals through reduced coenzyme Q_10_, and the two synergistically inhibit lipid peroxidation. MFHs can upregulate the expression of GPX4 and SLC7A11, downregulate the levels of ROS and TFR1, and inhibit ferroptosis.

Iron ion is an essential element for cell metabolism and growth and development. Iron deficiency or overload will affect the normal function of the body. Ferroptosis is a new type of programmed cell death characterized by iron dependence and lipid peroxidation. Its molecular mechanism mainly involves the inhibition of glutathione peroxidase 4 (GPX4) activity, iron metabolism disorder, and lipid peroxidation [24]. Studies have shown that serum proteomics in patients with depression show abnormal ferritin expression, while a chronic-stress-induced mouse depression model confirms that hippocampal iron deposition was significantly correlated with neuronal degeneration and death, suggesting that iron may be a potential biomarker for understanding the pathophysiological mechanism of depression [25,26]. In addition, ferroptosis may promote the occurrence of depression through a variety of ways; increasing the level of COX2 by upregulating the expression of the PTGS2 gene, so that the body tends toward an inflammatory state [27], dMT1-mediated induction of neuronal and neuronal damage, or downregulation of dopamine neurotransmitters through the PKC pathway [28]; it is also easier to increase the expression of ACSL4 and activate adrenal cortical cells, which then leads to an HPA axis disorder [29]. Therefore, the regulation of ferroptosis can be a feasible treatment strategy for depression and has great potential to promote further research and targeted drug development. The mechanism of MFHs in improving depression by reducing ferroptosis is shown in Figure 3.

### 2.6. Microorganism Gut–Brain Axis

**Figure 4:**
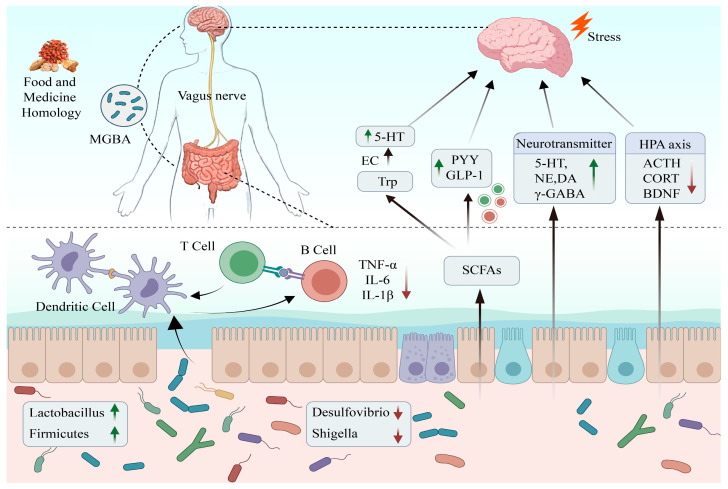
Antidepressant mechanism of MFHs targeted intervention in MGBA. Intestinal flora and their metabolites, such as SCFAs and tryptophan metabolites, transmit peripheral signals through the following three pathways: immunity, the vagus nerve, and the endocrine system. These signals affect the central HPA axis, neurotransmitters (GABA and 5-HT), and microglial function after crossing the blood–brain barrier. MFHs can regulate gut microbiota composition, repair the intestinal barrier, and promote the production of short-chain fatty acids and neurotransmitters.

The microbe gut–brain axis (MGBA) is a two-way communication pathway between the gut microbiota, the gut and the central nervous system. In recent years, studies have shown that intestinal flora can regulate the function of the central nervous system through the vagus nerve system, immune system, and endocrine system, thus affecting their mental state and behavior [30]. The intestinal microbial composition of patients with depression is significantly different from that of healthy people. The typical characteristics include the general decrease in the abundance of beneficial bacteria such as Lactobacillus and Bifidobacterium and the increase in the relative abundance of Bacteroidetes and Proteobacteria related to inflammation [31]. The specific mechanism of its action includes the following aspects: (1) Autonomic nervous system: the vagus nerve is a two-way signal transmission between the intestinal brain axis, which can not only transmit the brain’s signal to the intestine but also transmit the intestinal perception signal to the brain, affecting its emotional, cognitive, and other functions. (2) Immune system pathway: There are a large number of immune cells in the intestine, which constitute the immune barrier of the intestine. The increased intestinal permeability caused by the imbalance of the microbiota can promote inflammatory factors such as lipopolysaccharide (LPS) to enter the circulatory system, activate peripheral immune cells, and cause neuroinflammation [32]. (3) Endocrine system pathway: Intestinal microorganisms can directly synthesize or regulate serotonin, gamma-aminobutyric acid (GABA), and other neurotransmitters [33]; its metabolite short-chain fatty acids (SCFAs) can not only penetrate the blood–brain barrier to promote microglial maturation and synaptic plasticity but also regulate the activity of the HPA axis through the vagus nerve to alleviate stress response [34]. Therefore, MGBA has been gradually used as a new target for the treatment of depression. The mechanism of action of MFHs to improve depression through MGBA is shown in Figure 4.

## 3. Anti-Depressant Components in Medicinal and Food Homologous Substances and Their Mechanism of Action

**Figure 5:**
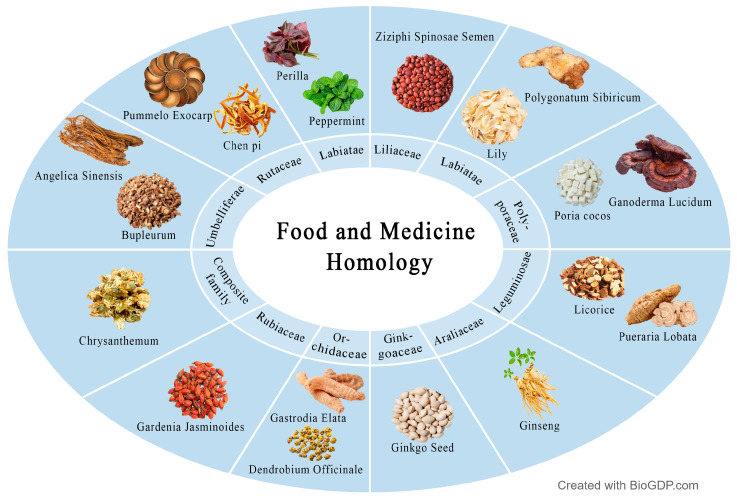
Distribution and representative components of main families and genera of MFHs with antidepressant potential.

As a special kind of dietary resource, MFHs are not only the carrier of functional components, but also the natural source of dietary nutrients. According to the statistics of Chinese Pharmacopoeia, there are more than 50 kinds of antidepressant traditional Chinese medicine used in clinical practice [35]. The distribution of main families and representative components are shown in Figure 5.

### 3.1. The Mechanism and Application of Dietary Nutrients

The prevention and treatment strategy of depression is gradually expanding from traditional intervention to the comprehensive path of nutrition and diet coordination. Studies have shown that specific nutrients such as omega-3 polyunsaturated fatty acids, folic acid, and mineral elements, including zinc, magnesium, and selenium, are closely related to the occurrence and development of depression [36]. China has a long history of food tonic health culture and rich dietary resources. By excavating the active ingredients with antidepressant potential in MFHs, clarifying their key nutrients and mechanism of action, and developing corresponding functional foods, this led to not only the modern inheritance of traditional food nutrition wisdom but also provides a new perspective and potential auxiliary intervention approach for the prevention and treatment of depression. As shown in Table 1, according to the Chinese food composition table, the dietary nutrient contents and antidepressant mechanisms related to depression in MFHs are summarized [37].

#### 3.1.1. Polyunsaturated Fatty Acids

As an important component of the nerve cell membrane, omega-3 fatty acids (ω-3PUFAs) are essential for brain function and are involved in visual, nervous system, intellectual development, and neurotransmitter metabolism. studies have shown that ω-3PUFAs can not only improve sleep and anxiety in patients with depressive disorders but also improve the cognitive function of people with depressive disorders [38]. Among the MFHs, *Perilla frutescens*, *Dolichos lablab*, and *Nelumbo nucifera* are rich in ω-3PUFAs; in addition, deep-sea fish, flaxseed, chia seed, walnut, and algae oil are also important sources of ω-3PUFAs in daily diet [39]. The mechanism of ω-3PUFAs improving depression involves multiple targets. For example, EPA is a precursor of derived anti-inflammatory active mediators, which can effectively inhibit the production of pro-inflammatory cytokines such as IL-6 and TNF-α [40]. It can also regulate monoamine neurotransmitters by affecting the physical properties of cell membranes and the endogenous cannabinoid system and increase the expression of BDNF in a cyclic adenosine monophosphate-independent manner [41]. In addition, clinical studies and animal experiments have found that ω-3PUFAs can improve the structure of intestinal flora and promote the production of short-chain fatty acids to exert antidepressant effects [42]. A large number of meta-analyses have also confirmed that supplementation of ω-3PUFAs can be regarded as an effective adjuvant therapy to alleviate depressive symptoms. Intake of 1 g of ω-3PUFAs per day and EPA content of 60.0% or more may improve depression [43].

#### 3.1.2. Folic Acid

Folic acid is a water-soluble B vitamin and a key nutrient for maintaining the health of the nervous system. It is rich in various MFHs such as *Cichorium intybus*, *Nelumbo nucifera*, *Lonicera japonica*, and *Citrus aurantium L.* var. *amara Engl.* Folic acid deficiency can lead to depression, dementia, fetal malformations, and other central nervous system problems. In terms of mechanism of action, folic acid is a key coenzyme for the synthesis of neurotransmitters such as 5-HT and DA, and participates in homocysteine metabolism as a methyl donor, which can reduce the high homocysteine level in the body, thereby reducing the neuroinflammation and oxidative stress induced by it, and reducing the risk of depression [44]. Clinical observations have confirmed that a considerable proportion of patients with depression have low folic acid levels or high homocysteine levels, so timely supplementation of folic acid or folic-acid-rich foods can help prevent the occurrence of depression [45].

#### 3.1.3. Mineral Elements

Mineral elements, such as zinc, magnesium, and selenium, are involved in a variety of biochemical pathways in the body, including nerve signal transmission and muscle relaxation, and are essential components of the human body. Zinc is present in synaptic vesicles in the cortex, hippocampus, and amygdala of the brain, and is released at any time to regulate brain zinc homeostasis. *Ostreidae*, *Zaocys dhumnades*, *Polygonum sibiricum*, *Cannabis sativa*, *Piper longum* and other substances are rich in zinc. Zinc can inhibit glutamate excitotoxicity by regulating NMDA receptors and activate GPR39 receptors to increase BDNF expression, thereby exhibiting antidepressant and neuroprotective properties [46]. As a natural blocker of NMDA receptor, magnesium is involved in hundreds of enzymatic reactions and can regulate the activity of HPA axis to reduce the stress response. Magnesium is more abundant in *Portulaca oleracea*, *Zaocys dhumnades*, *Glycine max*, *Houttuynia cordata*, *Cannabis sativa* and *Puerariae lobatae.* Dietary magnesium intake is negatively correlated with depressive symptoms. After magnesium supplementation, the anxiety level of rats is significantly reduced, and the neuroinflammation and synaptic enhancement of mice were normalized [47]. Selenium is a cofactor of various synthetic enzymes and metabolic enzymes. It has a high content in *Ginkgo biloba*, *Morus alba*, *Dimocarpus longan*, *Laminaria japonica* and *Sesamum indicum.* Selenium prevents lipid peroxidation and cell oxidative damage through selenoproteins such as glutathione peroxidase (GSH-Px) and thioredoxin reductase and plays a role in preventing inflammation and regulating neurotransmitters such as 5-HE and DA, which can prevent the risk of depression [48]. A clinical study found that high doses of selenium intake reduced the risk of depression by 54.0%, indicating that selenium has a potential antidepressant effect [49].

### 3.2. The Mechanism and Application of Active Functional Factors

#### 3.2.1. Flavonoids

Flavonoids are a class of polyphenolic secondary metabolites with a C6-C3-C6 structure. They are widely distributed in MFHs and are the most abundant and diverse sources of antidepressant active ingredients. From the existing research, Asteraceae, Moraceae, Rubiaceae, Leguminosae, Labiatae and other species are the main sources of a variety of highly active flavonoids. In addition, the structure–activity relationship study found that the antidepressant activity of flavonoids is closely related to the hydroxyl groups at specific positions on the parent nucleus, especially the 5th or 7th hydroxyl group of the A ring is considered to be the key pharmacophore [50]. For example, apigenin, quercetin, and luteolin all have this structural feature.

Flavonoids can reduce neuronal damage, inhibit neuroinflammation, and have antidepressant activity. Studies have shown that naringin extracted from *Citrus maxima* can play an antidepressant role by inhibiting inflammatory response and reducing apoptosis of hippocampal neurons [51]. Apigenin in *Perilla frutescens* significantly inhibits the release of inflammatory factors and the activation of NLRP3 inflammasome while regulating neurotransmitters such as 5-HT [52]. Quercetin is a common flavonol compound with significant antioxidant effect. It can also reduce the level of inflammatory factors in the serum of depressed mice, restore the expression of pCREB/BDNF/PSD95/Synapsin signaling pathway in the hippocampus, improve neuroplasticity, and, thus, alleviate depression [53]. In the regulation of ferroptosis, quercetin and luteolin can reduce lipid peroxidation and ferroptosis by upregulating the expression of core regulatory proteins such as SLC7A11 and GPX4 [54,55]. Icariin is a flavanol glycoside compound isolated from *Epimedium brevicornu*, which can promote the expression levels of estrogen receptor (ER) and ERα in the hypothalamus, and balance the sex hormone disorders in perimenopausal depression rats. And increase the expression levels of 5-HT, DA and NA and regulate the expression of PI3K-AKT-pathway-related proteins to enhance immune function [56]. Puerarin is the main isoflavone component in *Pueraria lobata*, which can effectively improve CUMS-induced depression-like behavior in mice, reshape the intestinal microflora, reduce pathogenic bacteria, such as Proteus, Spirillum, and Desulfovibrio, and increase the abundance of beneficial bacteria such as Firmicutes, Bacillus, and Lactobacillus [57].

The antidepressant effect of flavonoids involves a multi-target synergy, including the inhibition of neuroinflammation, anti-oxidation, promotion of neurotrophic and regulation of gut–brain axis, which reflects the overall regulation advantage of MFHs. Although its antidepressant potential is significant, the low bioavailability limits the neuroprotective effect, and nano-preparations and targeted delivery systems are expected to provide an improvement direction for this. Other flavonoids with significant antidepressant activity are shown in Table 2.

#### 3.2.2. Polysaccharides

Polysaccharide is a biologically active macromolecule composed of monosaccharides connected by glycosidic bonds. It is a key active component of many MFHs. Studies have shown that polysaccharides with antidepressant activity show obvious family and genus aggregation in MFHs. Among them, Polyporaceae, Liliaceae and Orchidaceae are the most concentrated sources of polysaccharides.

Polysaccharides in MFHs can exert antidepressant effects by regulating neurotransmitters, HPA axis, anti-inflammation, and regulating intestinal flora. For example, in the lipopolysaccharide-induced mouse depression model, Polygonati Rhizoma polysaccharide can significantly improve depression-like behavior, increase in the content of 5-HT and DA in the brain, reduce the level of pro-inflammatory cytokines in the hippocampus and the concentrations of CORT and ACTH in serum, indicating that its antidepressant mechanism is related to the regulation of neurotransmitters in the brain and a reduction in the HPA axis hyperfunction [63]. Astragalus polysaccharides can activate the Nrf2-ARE pathway in the hippocampus of depressed rats, increase in the levels of Nrf2 mRNA, Nrf2 total protein and nuclear protein in the hippocampus, and promote the activation and nuclear translocation of Nrf2. Astragalus polysaccharides can increase the activities of endogenous antioxidant enzymes, such as SOD, GSH-Px, CAT and HO-1 [64]. Lycium barbarum polysaccharides have also been shown to inhibit lipid peroxidation, enhance the antioxidant effect of striatum in depressed mice, and upregulate the expression of anti-apoptotic proteins Bcl-2 and PARP [65].

In addition, many polysaccharides themselves are excellent prebiotics that can be used by beneficial bacteria in the intestine. For example, Polygonati Rhizoma polysaccharide [63], Cistanche deserticola polysaccharide [66] and Ginkgo biloba polysaccharide [67] can effectively reduce the imbalance of intestinal flora caused by chronic stress, increase the abundance of beneficial bacteria such as Lactobacillus and Bacteroides, and promote the formation of short-chain fatty acids. By regulating the gut–brain axis, it reduces inflammation and relieves depression. Although many studies have revealed the antidepressant potential of polysaccharides, the current research on the structure-activity relationship is still limited. Most studies only analyzed monosaccharide composition, but failed to explore its structure-activity relationship. However, preliminary evidence has shown that structural units such as glucose, mannose, galactose and its uronic acid may play a key role in the antidepressant effect of polysaccharides, which deserves further attention in subsequent studies [68]. Other polysaccharides with significant antidepressant activity are shown in Table 3.

#### 3.2.3. Saponins

Saponins are a class of glycoside compounds with surface activity. Their molecular structure is composed of aglycones and sugar chains connected by glycosidic bonds. These compounds show obvious species distribution characteristics in MFHs: triterpenoid saponins are mainly distributed in the plants of Umbelliferae, Araliaceae and Rhamnaceae, while steroidal saponins are common in plants of Dioscoreaceae and Liliaceae. *Bupleurum chinense*, *Panax ginseng* and *Ziziphi spinosae semen* are regarded as representative raw materials with high saponin content, and their saponin components have become the research focus of antidepressant mechanism and product development.

In traditional Chinese medicine, *Bupleurum chinense* has the effect of soothing liver and relieving depression. It is often combined with other medicinal materials to form classical prescriptions such as Chaihu Shugan San and Xiaoyao San. Modern studies have shown that saikosaponin A significantly improved CUMS-induced behavioral deficits, its sucrose preference is significantly increased, the exercise time in the central region is increased, and the expression of 5-HT and NE in the hippocampus is increased [77]. Saikosaponin D reduces neuroinflammation by promoting the ubiquitination of NLRP3 and inhibiting the activation of inflammasomes [78]. Saikosaponin B2 mediates the TLR4-NFkβ pathway through GPX4, thereby reducing the occurrence of ferroptosis and neuroinflammation, showing the characteristics of multi-target antidepression [79]. Jujuboside regulates the concentration of monoamine neurotransmitters in the brain and regulates BDNF and Trk in depressed mice [80]. Dioscin can reduce the expression of inflammatory factors IL-6 and TNF-α, regulate the activity of HPA axis and increase the abundance of Firmicutes and Lactobacillus in stressed mice. It shows that dioscin has a systematic regulatory effect on intestinal flora composition, HPA axis and inflammatory response [81]. Gypenosides alleviate CORT-induced ferroptosis in PC12 cells by inhibiting the TNF-α/NF-κB signaling pathway, reducing the release of inflammatory factors, alleviating iron metabolism disorders and accumulation, and reducing lipid peroxidation [82]. *Panax ginseng*, as a traditional precious Chinese medicine, has been widely studied for its saponins. Ginsenoside Rg1 can reduce the degradation of neurotransmitters by inhibiting monoamine oxidase activity, regulate the expression levels of glucocorticoid receptor (GR), BDNF and its receptors, and improve nerve plasticity damage [83]. In addition, other saponins isolated from *Panax ginseng*, such as ginsenoside Rg3, Rb1, Re, Rh2, etc, have significant antidepressant activity [84]. Other antidepressant saponins are shown in Table 4.

#### 3.2.4. Terpenoids

Terpenoids are a class of natural secondary metabolites widely found in the plant kingdom. Their structures are constructed from isoprene (C5) as the basic unit. Among them, iridoid monoterpenes are the characteristic components of *Gardenia jasminoides* and *Cornus officinalis*, while triterpenes are mainly derived from Polyporaceae and Leguminosae plants. In addition, labiatae and ginkgo plants also contain specific terpenoids with antidepressant potential, which constitute antidepressant terpenoids with diverse structures and abundant sources.

Geniposide is a 7,8-cyclopentene type cyclic terpene compound, which is the main active ingredient in *Gardenia jasminoides*. As a glucagon-like peptide-1 receptor (GLP-1R) agonist, geniposide can inhibit the apoptosis of hippocampal neurons by activating the GLP-1R/AKT signaling pathway, reduce the levels of IL-1β and TNF-α, and improve the depressive symptoms of mice [91]. Chen et al. used serum metabolomics to find that geniposide can reverse the abnormalities of metabolites related to inflammatory response and glucose metabolism in serum and hippocampus of depressed mice, such as arachidonic acid and prostaglandin E2, and can also reduce the level of pro-inflammatory factors, thus exerting antidepressant effects [92]. Crocin is a kind of water-soluble carotenoids in *Gardenia jasminoides*, which can reduce inflammatory factors and increase BDNF protein expression, and alleviate depression-like behavior in mice [93]. In addition, a natural gardenia blue pigment formed by genipin and amino acid complexation also has antidepressant effect, which can regulate 5-HT, DA content and HPA-related hormone balance in rat brain, repair damaged hippocampal neurons, and show antidepressant activity [94]. Loganin is one of the main active ingredients in *Cornus officinalis*. It can increase the sucrose preference of CUMS model mice and the activity level in open field test (OFT). Its antidepressant mechanism is related to the regulation of neurotransmitters, HPA axis function and BDNF expression [95]. Catalpol is a key component of antidepressant in *Rehmannia glutinosa*, which can activate the transcription factor 3 (ATF3)/ferroptosis inhibitory protein 1 (FSP1) signaling pathway to reduce oxidative stress, reduce intracellular Fe^2+^ ion content, and inhibit ferroptosis [96]. Glycyrrhizic acid and glycyrrhetinic acid are the most studied triterpenoid saponins in *Glycyrrhiza uralensis*, and their antidepressant effects have been found to be related to high mobility group protein B1 (HMGB1) and kynurenine pathway [97]. Other terpenoids with significant antidepressant activity are shown in Table 5.

## 4. Development Status of Medicine and Food Homologous Products for Relieving Depression

In recent years, with the enhancement of national health awareness, MFHs with both traditional health wisdom and modern health concepts are increasingly being used in adjuvant treatment to improve depression and anxiety, and the related functional food market is also showing a booming trend.

The product development of MFHs in depression mainly focuses on raw materials with the effects of soothing liver and relieving depression, tranquilizing mind, invigorating spleen and replenishing qi. Through statistical analysis via data mining, the high-frequency used MFHs mainly include *Poria*, *Angelica sinensis*, *Glycyrrhiza uralensis*, *Citrus reticulata*, *Ziziphi spinosae semen*, *Gardenia jasminoides*, *Astragalus membranaceus*, *Lilium brownii*, *Cornus officinalis*, etc. [104]. These substances are mainly warm and flat in medicinal properties, with sweet, pungent and bitter tastes as the core, mainly belonging to the three meridians of liver, heart and spleen, reflecting the core pathogenesis of ‘liver and spleen treatment’ in traditional Chinese medicine. In the application of compatibility, ancient and modern prescriptions follow the law of ‘monarch, minister, assistant and envoy’, and form a number of core drug pairs and prescriptions. For example, ‘*Poria-Angelica sinensis*’ is used for depression with spleen deficiency and blood deficiency, and ‘*Gardenia jasminoides-Glycine max*’ is used for depression with blood stasis and liver depression [105]. In terms of product form, it gradually shows a trend of diversification and innovation. On the one hand, based on the classic prescriptions such as Xiaoyao Powder and Ganmai Dazao Decoction, granules, capsules, tablets and other forms are derived as drug forms; it is also widely developed into functional foods such as solid beverages, substitute teas, fermented beverages and pastes. For example, there have been solid drinks containing *jujube kernel*, *poria cocos* and other ingredients on the market, as well as portable tea drinks containing *bergamot*, *rose* and other ingredients. These products are usually claimed to help improve sleep, relieve stress and other effects [106,107,108]. These products are usually claimed to help improve sleep, relieve stress and other effects. Consumers can indirectly regulate physical function and emotional state through daily diet, but its antidepressant effect is mainly based on traditional application experience and animal experimental evidence, and there is still a lack of strict clinical verification. In the future, the evaluation of product efficacy should be strengthened to promote the standardized development of medicinal and food functional foods so as to better meet the market demand for emotional health dietary intervention [109].

## 5. Application of Modern Technology in the Development of Medicine and Food Homology to Improve Depression

In the modern processing system of MFHs, the pretreatment of raw materials directly affects the subsequent processing effect and the biological activity of the final product [110]. Traditional processing methods (such as steaming, boiling, stir-frying and stir-frying) mainly rely on exogenous heat, force or medium to achieve the purpose of reducing toxicity, modifying and promoting the dissolution of specific components [111]. In the development of modern health products, microbial fermentation technology shows unique potential because it can improve the content of active ingredients and promote the generation of new active substances. This functional optimization based on biotransformation not only lays a foundation for the development of efficient antidepressant products, but also provides a clear research and development path for the subsequent construction of brain-targeted nano-delivery system and the use of multi-omics technology to systematically analyze the synergistic mechanism between component-target-pathway.

### 5.1. Research Status of Microbial Fermentation of MFHs

As a key means of processing MFHs, microbial fermentation technology has shown significant advantages in improving medicinal value, reducing toxic and side effects, and promoting the transformation of active ingredients in recent years. This technology uses targeted strains such as Lactobacillus, yeast or Aspergillus to carry out solid-state, liquid-state or two-way fermentation of MFH raw materials. The active ingredients such as macromolecular polysaccharides, flavonoids, and saponins are converted into small molecular active substances that are more easily absorbed by the enzymes produced by microbial metabolism to break the plant cell wall. At the same time, prebiotics, probiotics, γ-aminobutyric acid, short-chain fatty acids and other metabolites with neuroregulatory effects are enriched [112]. Fan et al. found that lactic acid bacteria-fermented ginseng can convert its main saponin Rb1 into more active rare saponins (such as Rg3 and Rh1), and significantly reduce LPS-induced inflammatory response in mice by inhibiting TLR4/MAPK signaling pathway and enhancing intestinal barrier function, which is better than unfermented ginseng [113]. Sun Lu et al. found that the Suanzaoren products fermented by Poria cocos and Shenqu can improve the sedative and sedative effects and improve insomnia. The mechanism may be related to the regulation of monoamine neurotransmitters in the brain [114]. Li et al. found that the Polygonatum polysaccharide fermented by Lactobacillus plantarum could significantly regulate the intestinal flora of mice and improve the inflammatory indexes, while patients with depression were often accompanied by intestinal floral imbalance [115]. Therefore, microbial fermentation not only improves the bioavailability of functional components but also regulates neuroendocrine and immune systems through the gut–brain axis pathway, providing multi-channel intervention potential for improving depression.

In addition, microbial fermentation can also be used for secondary utilization of MFHs waste residue for feed, or develop functional foods, such as fruit wine, fruit vinegar and lactic acid drinks, with anti-oxidation and regulation of intestinal flora. In the future, multi-omics technology can be used to analyze the relationship between flora, metabolism and efficacy in the fermentation process and to establish a standardized process and quality system so as to give full play to the unique advantages of MFHs in the field of mental health.

### 5.2. Study on Nano-Delivery System in MFHs

In the development of MFHs, the stability and bioavailability of functional components are the key factors to determine their clinical application effects. Many natural active ingredients are easily degraded or inactivated during extraction and storage, resulting in low bioavailability and limited stability [116]. The presence of blood–brain barrier further limits their distribution and efficacy in brain tissue [117]. In order to overcome the above difficulties, nano-drug delivery systems have attracted extensive attention in recent years. With the help of carriers such as liposomes, polymer nanoparticles, micelles, and plant exosome-like nanoparticles, active ingredients can be encapsulated or loaded [118]; and their delivery efficiency and brain distribution can be improved by improving solubility, delaying release, and enhancing targeting [119]. For example, CS BSA nanocarriers prepared with chitosan and bovine serum albumin not only improve the penetration ability of curcumin across the blood–brain barrier, but also exhibit anti-inflammatory and neuroprotective effects by inhibiting M1 macrophage polarization and blocking TLR4-mediated MAPK/NF-κB signaling pathway [120]. Huang et al. enhanced the thermal stability and antioxidant activity of luteolin by coating liposomes containing luteolin with whey protein isolate [121]. These studies have shown that nano-delivery systems can not only improve the bioavailability and brain targeting efficiency of MFHs but also may produce synergies with their loaded components through the biological characteristics of the carrier itself, thus providing a new way for the development of neuroprotective agents with both efficient delivery and multi-target intervention functions.

### 5.3. Multi-Omics Technology Reveals the Molecular Mechanism of Antidepressant Effect of MFHs

Medicinal and edible monomers and compounds usually contain multiple active ingredients, and their antidepressant effects have the characteristics of multi-target synergy. With the development of molecular biology, genomics and modern pharmacology, multi-omics techniques such as transcriptomics, proteomics and metabolomics are increasingly used to reveal the mechanism of the effects of MFHs on the nervous system. Proteomics can identify disease markers and elucidate the mechanism of drug action by analyzing protein composition and abundance changes. For example, Zeng et al. identified 52 differentially expressed proteins in cerebrospinal fluid of CUMS depression model rats, involving ribosome and the PI3K/Akt and IL-17 signaling pathways, indicating that icariin may improve depressive symptoms and hippocampal neurogenesis by regulating key proteins in cerebrospinal fluid [122]. Dong et al. used iTRAQ technology to study and found that 33 differentially expressed proteins were identified in the hippocampus of CUMS rats after Kaixinsan intervention, which were involved in synaptic plasticity and neurogenesis. Metabolomics, by systematically analyzing the overall changes of endogenous small molecule metabolites, reveals the metabolic pathways related to the homology of medicine and food, screens specific biomarkers, and clarifies its mechanism of action [123]. For example, GU et al. found that Acanthopanax senticosus can exert antidepressant effects by affecting glycine, serine and threonine metabolism and starch and sucrose metabolism through network pharmacology and metabolomics studies [124]; xiaoyaosan exerts its efficacy by regulating key metabolites such as arachidonic acid metabolism and N-acetylaspartate [125]. However, the dimension of single omics analysis is limited, and the combination of multi-omics can more accurately and comprehensively reveal its mechanism of action and evaluate the toxicity of traditional Chinese medicine. In addition, multi-omics technology can be combined with network pharmacology and molecular docking technology, and with the help of artificial intelligence and machine learning algorithms, the interaction between active ingredients and targets can be predicted, compound compatibility can be optimized, and its pharmacodynamics can be simulated, thus providing an important reference for elucidating the potential neuroprotection, regeneration and repair effects of plant components.

## 6. Conclusions and Prospect

As a common mental illness, the prevention and treatment of depression has been included in the important category of ‘Healthy China’ action. The pathogenesis of the disease is complex, involving neurotransmitter disorders, HPA axis dysfunction, neuroinflammation and other pathways, and individual differences are significant. In recent years, studies on the mechanism of ferroptosis and microbial gut–brain axis have provided a new perspective for understanding the systemic pathology of depression and expanded the intervention targets. In this context, MFHs with multi-target and multi-pathway regulation characteristics show unique advantages. In this paper, more than 40 kinds of MFHs with antidepressant potential are sorted out, which are widely distributed in Leguminosae, Liliaceae, Araliaceae, Compositae, Labiatae and Polyporaceae. They are not only rich in functional active ingredients, especially flavonoids, polysaccharides, saponins and terpenoids but also play an antidepressant role through multi-target and multi-pathway mechanisms. It also contains conventional dietary nutrients (such as omega-3 polyunsaturated fatty acids, folic acid and mineral elements), and its deficiency increases the risk of depression, which can be supplemented safely and conveniently through daily diet. In addition, by optimizing dosage forms and consumption scenarios, it can be transformed into solid beverages, substitute tea, snacks and fermented functional beverages, which are more in line with the rhythm of modern life. It is an important path to realize the daily application of medicine and food homology.

Although MFHs have shown great potential in the treatment of depression, most of the current research evidence comes from animal experiments (such as CUMS, LPS, and CORT induction models) and in vitro cell experiments, and there is still a lack of large-scale randomized controlled clinical trials. Although animal models can simulate some depressive phenotypes, they are still different from clinical practice. The oral bioavailability of most functional components is low, and the blood–brain barrier penetration is limited, which limits their clinical transformation value to a certain extent. In addition, compared with the widely used antidepressants, most of the research on MFHs remains in the preclinical stage, and their exact antidepressant effect and long-term safety need to be further verified.

Therefore, in the future, clinical exploratory experiments should be strengthened and combined with microbial fermentation, nano-delivery and other technologies to enhance targeting and therapeutic effects, and multi-omics and network pharmacology should be used to deepen the mechanism research so as to promote the precise application of MFHs in dietary intervention of depression.

## Figures and Tables

**Table 1:** Main nutritional components of representative MFHs.

Nutrient Category/Content Unit	Representative Medicine and Food Homology	Content	Antidepressant Mechanism
polyunsaturated fatty acid (g/100 g)	*Perilla frutescens*	50.16	1. Anti-neuroinflammation 2. Promoting neurotrophy 3. Regulating intestinal flora
	*Dolichos lablab*	11.0	
	*Nelumbo nucifera*	2.1	
	*Morus alba*	1.5	
	*Ziziphus jujuba*	0.9	
folic acid (μg/100 g)	*Cichorium intybus*	1546.77	1. Involved in the synthesis of a variety of coenzymes and neurotransmitters 2. Increase the content of 5-HT 3. Participation in energy metabolism
	*Nelumbo nucifera*	1122.43	
	*Lonicera japonica*	400.87	
	*Citrus aurantium L.* var. *amara Engl.*	309.11	
	*Houttuynia cordata*	221.49	
	*Sophora japonica*	182.3	
zinc (mg/100 g)	*Ostreidae*	52.17	1. NMDA receptor regulation 2. Promoting neurotrophic 3. Regulating 5-HT production
	*Zaocys dhumnades*	13.55	
	*Polygonum sibiricum*	10.58	
	*Cannabis sativa*	10.34	
	*Piper longum*	14.15	
magnesium (mg/100 g)	*Portulaca oleracea*	3154.6	1. NMDA receptor blockade 2. Regulating HPA axis 3. Energy generation
	*Zaocys dhumnades*	3106.0	
	*Glycine max*	1258.0	
	*Houttuynia cordata*	960.3	
	*Cannabis sativa*	651.8	
	*Puerariae lobatae*	615.0	
selenium (μg/100 g)	*Ginkgo biloba*	14.5	1. Antioxidation 2. Regulating neurotransmitters 3. Anti-neuroinflammation
	*Morus alba*	34.0	
	*Dimocarpus longan*	12.4	
	*Laminaria japonica*	5.84	
	*Sesamum indicum*	4.70	

**Table 2:** Antidepressant components and mechanism of flavonoids.

Classification	Active Ingredient	Source	Experimental Model	Dosage	Mechanism	Reference
Flavonoids	Naringin	*Citrus maxima*	LPS-induced depression mouse model	80 mg/kg	Inhibits inflammatory response and reduces apoptosis of hippocampal neurons	[51]
	Apigenin	*Perilla frutescens*	CMS rat model	20 mg/kg	Increased 5-HT concentration, decreased 5-HIAA and DA concentration, inhibited IL-1β production and NLRP3 activation	[52]
	Luteolin	*Lonicera japonica*, *Chrysanthemum morifolium*	CUMS mouse model	10, 20, 30, 40 mg/kg	Through IRF1/SLC7A11/GPX4 signaling pathway reduces inflammatory polarization, lipid peroxidation and ferroptosis	[54]
Flavonols	Quercetin	*Sophora japonica*	LPS-induced depression mouse model	10 mg/kg	Inhibits activation of the PI3K/AKT/NF-κB inflammatory signaling pathway and improves neuroplasticity	[53]
			Perimenopausal depression rat model	50 mg/kg	Increases the expression of GPX4 and SLC7A11	[55]
	Isorhamnetin	*Hippophae rhamnoides*	CUMS mouse model	20, 100 mg/kg	Increasing the contents of neurotrophic factors and neurotransmitters and changing the diversity of intestinal flora	[58]
	Myricetin	*Morus alba*, *Ginkgo biloba*	SPS rat model	10, 20 mg/kg	Regulating the HPA axis and activating the BDNF-ERK signaling pathway	[59]
	Hyperoside	*Apocynum venetum*	CUMS mouse model	2.5, 5 mg/kg	Increases the concentration of zinc and BDNF in the hippocampus of mice	[60]
	Icariin	*Epimedium brevicornu*	Perimenopausal depression rat model	12.5, 25, 50 mg/kg	Balances the sex hormone disorder in depressed rats, regulate the secretion of neurotransmitters and enhance immune function	[56]
Dihydroflavone	Liquiritin	*Glycyrrhiza uralensis*	CUMS mouse model	20~40 mg·kg^−1^	Inhibition of NLRP3 inflammasome-mediated inflammatory response	[61]
Isoflavone	Puerarin	*Pueraria lobata*	LPS-induced depression mouse model	50, 100, 200 mg/kg	Increases the content of SCFAs in intestinal flora and inhibit the activation of hippocampal microglia	[57]
Flavanones	Hesperidin	*Citrus reticulata*	CUMS mouse model	50, 100, 200 mg/kg	Inhibiting NCOA4-ferritin phagocytosis and alleviating dendritic spines in model mice	[62]

**Table 3:** Ingredients and mechanisms of antidepressant effects of polysaccharides.

Active Ingredient	Monosaccharide Composition	Experimental Model	Dosage	Mechanism	Reference
Polygonatum sibiricum polysaccharides	Man, Glc, Gal, Fru	CUMS mouse model	100, 200, 400 mg/kg	Inhibits HPA axis overactivation and neuroinflammation and regulates neurotransmitter levels and intestinal flora composition	[63]
Cistanche deserticola polysaccharides	Glc, Gal, Rha, Ara, Fru	CUMS rat model	200 mg/kg	Improves the abundance of beneficial bacteria and increases the content of SCFAs; maintains the balance of amino acid metabolism	[66]
Angelicas inensis polysaccharides	Glc, Gal, Ara, Rha, Fuc, Xyl, GalA	CUMS mouse model	20, 40 mg/kg	Enhances the synthesis of 5-HT, DA and GABA/GLU ratio and regulates neurotransmitter conduction	[69]
Poria cocos polysaccharides	Glc, Gal, Man, Ara	LPS-induced mouse model, BV2 cells	20, 80 mg/kg	The levels of ROS, NO, TNF-α and IL-1β in BV-2 cells decreased, and the NF-κB and NLRP3 signaling pathways were inhibited	[70]
Ganoderma lucidum polysaccharides	Glc, Ara, Xyl, Man, Gal	CSDS mouse model	1, 5, 12.5 mg/kg	The expression of BDNF, IL-1, GluA1 and GluA2 in hippocampus was upregulated, and the levels of IL-5β and TNF-α decreased	[71]
Lycium barbarum polysaccharide	Ara, Rha, Xyl, Man, Gal, Glc	Lps-induced depression mouse model	80 mg/kg	Reduces lipid peroxidation and enhances the antioxidant effect and the expression of anti-apoptotic proteins Bcl-2 and PARP	[65]
Gastrodia elata polysaccharide	Glc, Man, Fru	CUMS + LPS-induced mouse model	50, 100, 200 mg/kg	Restoring gut microbiota balance, activating the Keap1-Nrf2/BDNF-TrkB pathway, and enhancing antioxidant capacity and synaptic plasticity	[72]
Astragalus polysaccharide	Glc, Gal, Ara, GalA, Rha, Man	CUMS rat model	200, 400 mg/kg	The expression levels of SOD, GSH-Px, CAT and HO-1 increased, and the content of MDA decreased.	[64]
Ginkgo biloba polysaccharide	Man, Rha, GlcA, Gal, Ara	CUMS mouse mode	300 mg/kg	Increases levels of 5-HT and DA, improves the intestinal microbial imbalance in mice, and increases the richness of Lactobacillus species	[67]
Dendrobium officinale polysaccharides	Rha, Ara, Fuc, Man, Glc	Ovariectomy + CMS induced mouse model	150, 300, 600 mg/kg	It can reduce the levels of CRH, ACTH and CORT in serum, restore HPA axis, reverse neuroinflammation and regulate intestinal flora	[73]
Yam polysaccharide	Rib, Rha, Ara, Xyl, Man, Glc, Gal	H_2_O_2_-Induced Oxidative Damage in IEC-6 Cells	200, 400, 800 μg/mL	Inhibition of the MAPK pathway reduces oxidative damage in cells	[74]
Lily polysaccharide	Gal, Glc, Rha, Ara	CUMS mouse mode	200 mg/kg	The protein levels of ADCY6, PKA, CREB-1 and BDNF were upregulated by regulating the HPA axis	[75]
Lonicera japonica polysaccharide	GalA, Rha, Gal, Ara, Glc, Man	CUMS mouse model	30, 100 mg/kg	Inhibition of NLRP3 inflammasome-mediated immune inflammatory response	[76]

Mannose: Man; glucose: Glc; galactose: Gal; fructose: Fru; rhamnose: Rha; arabinose: Ara; fucose: Fuc; xylose: Xyl; galacturonic acid: GalA; glucuronic acid: GlcA; ribose: Rib.

**Table 4:** Ingredients and mechanism of the antidepressant effect of saponins.

Classification	Active Ingredient	Source	Experimental Model	Dosage	Mechanism	Reference
Triterpenoidal saponin	Saikosaponin A	*Bupleurum chinense*	CUMS rat model	12.5, 25, 50 mg/kg	Increases the expression of 5-HT, NE and other neurotransmitters and antioxidant enzyme activity, reduces inflammation	[77]
	Saikosaponin D		CUMS mouse model	8 mg/kg	Promotes the ubiquitination of NLRP3, inhibits the activation of inflammasome and improves inflammation	[78]
	Saikosaponin B2		CUMS + ferritin deposition mouse model	10 mg/kg	Inhibition of TLR4/NF-kβ pathway-mediated ferritin deposition and microglial activation	[79]
	Ginsenoside Rg1	*Panax ginseng*	CRS rat model	5, 10 mg/kg	Regulates neurotransmitter levels, increases antioxidant enzyme activity and restores BDNF-TrkB signaling in the prefrontal cortex	[83]
	Ginsenoside Rg3		SPS rat model	25, 50 mg/kg	Regulates the HPA axis and increases the expression of BDNF and TrkB mRNA in the brain	[85]
	Ginsenoside Rb1		CUMS rat model	5 mg/kg	Glu increased the levels of 5-HT, 5-HIAA, NE, DA and GABA, and decreased the level of Glu	[86]
	Ziziphi spinosae semen saponins	*Ziziphi spinosae semen*	CORT-induced depression mouse model	110 mg/kg	The contents of NE, DA and 5-HT in hippocampus and frontal cortex increased	[80]
	Astragaloside IV	*Astragalus membranaceus*	CUMS rat model	40 mg/kg	Increasing the abundance of beneficial bacteria, regulating the imbalance of Th17/Treg cells and the abnormal content of pro-inflammatory factors	[87]
	Gypenosides	*Gynostemma pentaphyllum*	Corticosterone induced PC12 cell injury	150 mg/mL	Inhibition of GLS2 expression, upregulation of SLC7A11 and GPX4 expression, reduction in glutamate accumulation and GSH consumption	[82]
	Mogroside V	*Siraitia grosvenorii*	CUMS rat model	10, 30 mg/kg	Inhibits inflammation and oxidative stress and reduces hippocampal neuronal apoptosis	[88]
Steroidal saponin	Diosgenin	*Dioscorea polystachya*	CUMS mouse model	20, 40, 80 mg/kg	Reduces CORT content and increases BDNF and 5-HT content; reduces the content of MDA and increases the content of SOD and CAT	[81]
	Polygonatum saponins	*Polygonatum sibiricum*	SPS-induced mouse model	20, 40, 60 mg/kg	The contents of 5-HT, NE and DA in prefrontal cortex and hippocampus of mice increased	[89]
	Lily saponins	*Lilium brownii*	CUMS mouse model	50, 100 mg/kg	Reduces serum CORT levels, regulates the BDNF/AKT/mTOR signaling pathway, and improves synaptic plasticity	[90]

**Table 5:** Ingredients and mechanism of antidepressant effect of terpenoids.

Classification	Active Ingredient	Source	Experimental Model	Dosage	Mechanism	Reference
Monoterpenes	Geniposide	*Gardenia jasminoides*	CMS rat model	/	Regulates inflammation-related metabolic pathways and glucose metabolism reduces the level of inflammatory factors.	[92]
			RRS-induced depression model in mice	50, 100 mg/kg	Regulates the glucagon-like peptide 1 receptor (GLP-1R)/protein kinase B/(AKT) signaling pathway	[91]
	Genipin		CUMS rat model	25, 50 mg/kg	Down-regulates apoptosis-related proteins and improve neuronal apoptosis	[98]
	Loganin	*Cornus officinalis*	CUMS mouse model	12.5, 50 mg/kg	The levels of ACTH and CORT decreased, the levels of neurotransmitters increased, and the expression of BDNF was promoted	[95]
	Catalpol	*Rehmannia glutinosa*	CUMS mouse model	10, 20 mg/kg	Activation of ATF3/FSP1 signaling pathway inhibits ferroptosis	[96]
	Aucubin	*Eucommia ulmoides*	CUMS mouse model	10, 20 mg/kg	Regulates HPA axis hormone levels, inhibits NF-κB/NLRP3 pathway, and reduces IL-1β and IL-18 expression levels	[99]
	L-menthone	*Mentha haplocalyx*	CUMS mouse model	15, 30 mg/kg	Anti-neuroinflammation, restoration of 5-HT and NE levels in the prefrontal cortex	[100]
Triterpenes	Ganoderic acid A	*Ganoderma lucidum*	LPS-induced mouse model	2.5, mg/kg	Inhibition of caspase-1 activity and inflammatory cytokines.	[101]
	Asiaticoside	*Centella asiatica*	CMS mouse model	20, 40 mg/kg	It may inhibit NF-κB and NLRP3-related inflammation by activating cAMP/PKA signaling pathway	[102]
	Glycyrrhizic acid	*Glycyrrhiza uralensis*	CUMS mouse model	20 mg/kg	Inhibit the expression of HMGB1 and reduce neuroinflammation	[97]
Diterpenes	Ginkgolide	*Ginkgo biloba*	CUMS rat model	5.4 mg/kg	Activation of neurotrophin-related NT3-TrkA pathway and neuroplasticity-related Ras-MAPK pathway	[103]
	Crocin	*Gardenia jasminoides*	Post-stroke depression rats	50 mg/kg	Reduces inflammatory factors, increases BDNF and SCFAs levels, and regulates intestinal flora imbalance	[93]

## Data Availability

The original contributions presented in the study are included in the article, further inquiries can be directed to the corresponding author.

## Abbreviations

The following abbreviations are used in this manuscript:

CUMS	Chronic unpredictable mild stress
SPS	Post-traumatic stress disorder
CSDS	Chronic social defeat stress
CRS	Chronic restraint stress
CORT	Corticosterone
RRS	Repeated restraint stress
EPA	Eicosapentaenoic acid
